# Internet Use Influences Self-Related Process: Evidence From Behavior and ERPs

**DOI:** 10.3389/fpsyg.2018.02597

**Published:** 2018-12-18

**Authors:** Gai Zhao, Yan Zhang, Fanchang Kong, Zhaojun Liu, Yadan Wang, Bo Zhou, Xingjie Zhang, Feng Tang, Zongkui Zhou

**Affiliations:** ^1^School of Psychology, Central China Normal University, Wuhan, China; ^2^Key Laboratory of Adolescent Cyberpsychology and Behavior, Ministry of Education, Wuhan, China; ^3^School of Education, Huazhong University of Science and Technology, Wuhan, China

**Keywords:** Internet use, self-related stimuli, implicit priming task, N2, LPC

## Abstract

The present study aimed to examine whether a self-related stimulus produces a self-related process bias between pathological-tendency Internet users and ordinary Internet users. Participants were asked to judge the color of the target stimulus’ frame (Internet pictures) in an implicit priming task, which enclosed the prime of self/other related words and the target of the online image in sequence. Results from Experiment 1 showed that response time (RT) in the self-related condition was significantly longer than that of the other related condition. Further analysis showed that RT in the self-related condition was significantly longer than that under the other related conditions for pathological-tendency Internet users but not for ordinary Internet users. In Experiment 2, behavior results demonstrated that RT under the self-related condition was significantly longer than that in the other-related condition for both groups, and the RT was shorter for pathological-tendency Internet users than that of the ordinary Internet users. Moreover, ERP data showed that the N2 amplitude was larger in the self-related condition than that of other related conditions for pathological-tendency Internet users but not for ordinary Internet users. The amplitudes of late positive component (LPC) was smaller in the self-related condition than those of the other related conditions. Hence, the Internet use influenced the inhibition control in self-unrelated stimuli and automatically retrieved the self-related stimuli.

## Introduction

According to a world survey on the Internet, the number of Internet users exceeded 3.5 billion or almost half of the world population in 2017 and has increased steadily in subsequent years. Young people are more likely to be online than their elders (International Telecommunication Union [ITU], 2017). In China, the number of netizens reached 772 million in December 2017 with an Internet penetration rate of 55.8% (China Internet Network Information Center [CNNIC], 2018). Moreover, in China, 97.5% of the netizens accessed to the Internet via cellphone, whereas 53.0, 35.8, and 27.1% of the netizens accessed to the Internet via desktop computer, laptop, and tablet computer, respectively, in December 2017 (China Internet Network Information Center [CNNIC], 2018). Clearly, the penetration rate of cellphone and computer is relatively high. Internet is a twofold sword that is highly beneficial to our lives. However, it also causes issues on problematic psychology and behaviors (Liu et al., 2012; De Leo and Wulfert, 2013; Mok et al., 2014).

Pathological Internet use is characterized by excessive and compulsive preoccupation with the Internet and loss of control over its use (Davis, 2001; Caplan, 2002; van den Eijnden et al., 2008). Pathological Internet use may affect many aspects of individuals’ cognition processes (He et al., 2008; Sun et al., 2009; Pawlikowski and Brand, 2011; Näsi and Koivusilta, 2013). A behavioral study showed that the Internet addicts were impaired in the executive control function in the Stroop task (Dong et al., 2011). Another behavior study used Edwardian gambling task (IGT) to confirm that the decision-making ability of the Internet addiction decreased; specifically, the pathological Internet users’ time of selecting strategy was longer than the control group (Sun et al., 2009). Evidence from the ERP studies showed that the P300 amplitude of online game addiction was significantly lower than that of non-addiction, indicating that pathological Internet users decreased in cognitive ability (He et al., 2008). Moreover, other ERP studies also verified that overusing the Internet reduced the individual’s P300 amplitudes and increased the P300 latency (Yu et al., 2008, 2009). An fMRI study illustrated that long-time online game playing enhanced the brain synchronization in sensory-motor coordination-related brain regions but decreased the excitability in visual- and auditory-related brain regions (Dong et al., 2012). Another fMRI study showed that Internet addicts manifested higher superior temporal gyrus activations and brain activation in bilateral insula than healthy subjects. Therefore, we conclude that Internet addicts engaged more endeavors in executive control and attention in the switching task. From another perspective, Internet addicts showed impaired cognitive flexibilities (Dong et al., 2014).

Self-related process is an advanced cognition process; studies on the self-related process have attracted many psychologists from the 1970s (Rogers et al., 1977). Northoff et al. (2006) collectively defined self-related, self-relevant, and self-reference processes as self-reference processing, and these concepts emphasized the correlation degree of stimuli and self. Self-reference can facilitate recording and refining stimuli related to one’s self-concept better than those of others (Rogers et al., 1977; Zhu, 2004; Kim, 2012). A multitude of studies have investigated the self-related process, including the stimuli of face (Northoff et al., 2006; Keyes et al., 2010), name (Chen et al., 2011; Tacikowski et al., 2011; Fan et al., 2016), birthday month (Rathbone and Moulin, 2010), and self-relevant possessive pronoun (Zhou et al., 2013). In the present study, we adopted self-related words (e.g., self) and other related words (e.g., other) as priming materials (He and Zhu, 2016).

Behavioral research has shown that people tend to perceive their pictures or close friends more accurately than completely unknown people do (Northoff et al., 2006). When the birthday of a target is the same as that of a subject, the recall accuracy ratio for the subject’s birthday is higher than that of other subjects’ birthdays (Brédart et al., 2013). For neural correlates, one ethnic label yields smaller N2 amplitudes than other ethnic labels do in an Oddball task (Wang et al., 2015). N2 is related to the early processing of self-relevant information (Wang et al., 2015). LPC is also one of the indexes of the self-reference effect. LPC is related to the sustained attention and stimulation coding (Kok, 1997), indicating the continuous input of cognitive resources and information storage (Fields and Kuperberg, 2016). LPC usually begins from 400–500 ms of stimulus onset and continues for hundreds of milliseconds (Friedman and Johnson, 2000). Kotlewska and Nowicka (2016) reported a greater LPC in processing past self than that of celebrities. In the context of career choice, the LPC of a self-related stimulus is greater than that of other stimuli (Zhong et al., 2014). Self-related stimuli eliciting strong responses are related to the continuous assignment of attention resources to self-related information (Kotlewska and Nowicka, 2016).

Although the self-related process and the pathological Internet use were extensively studied separately, the study on how the pathological Internet use influences the cognition process of individual self-related information is poorly explored. Yang et al. (2012) found that similar cognition process existed between real self and virtual self for ordinary people. A recent study examined the relationship between the Internet use and self-related information processing (Chen and Chen, 2017). The accuracy ratio of the real self was higher than that of the virtual self in the contrast group, but not for the Internet overuse group, suggesting that the Internet overuse processed self-related information more frequently in the Internet activities as compared with the contrast group (Markus, 1977; Chen and Chen, 2017). The above-mentioned studies did not consider the condition in the pathological Internet use, possibly affecting the self-related information more strongly. Therefore, the present study aimed to investigate the difference of self-related information process between pathological Internet-tendency users and ordinary Internet users.

College students account for a high portion of the population of Internet users. A national survey reported that up to 25.4% Chinese netizens are college students, the highest among the groups (China Internet Network Information Center [CNNIC], 2018). College students are in an important stage of active self-exploration and maturity. Thus, frequent and continuous immersion in the virtual world likely impairs their cognition ability, including self-related processing. Pathological Internet users have more Internet use experience than ordinary Internet users (Liu et al., 2015). Thus, pathological Internet users differ from ordinary Internet users in processing self-related stimuli. However, the behavioral evidence of the influence of the Internet use on self-related information was previously studied. To describe the full picture, we would further examine the influence of the Internet use on the self-related process at behavioral (Experiment 1) and neural (Experiment 2) levels in the prime task.

## Experiment 1

### Method

#### Participants

A sample of 300 college students was recruited for the survey of pathological Internet use. According to Lei and Yang (2007), subjects scored more than 120 were grouped as pathological-tendency Internet users, subjects scored less than 120 were grouped as ordinary Internet users (Lei and Yang, 2007). Finally, 19 pathological-tendency Internet users (*M* = 130.79, *SD* = 9.35), 23 ordinary Internet users (*M* = 94.17, *SD* = 14.56) were recruited in Experiment 1. Participants aged 18–25 years (*M* = 19.64, *SD* = 1.61), and were healthy, right-handed, with normal or corrected to normal vision, and self-reported no history of brain injuries or affective disorders. Each participant was paid RMB 20 Yuan after they completed the experiment. The present study was approved by the Central China Normal University Human Ethics Committee for Behavior Research, and written informed consent were obtained from all participants before experiment.

#### Measurements

##### Adolescent pathological Internet use scale (APIUS)

Developed by Lei and Yang (2007), APIUS was used to measure levels of pathological Internet use. It comprised 38 items with 6 dimensions, namely salience (e.g., I forget nearly everything else when I am online), tolerance (e.g., I find that I increasingly spend time online), withdrawal symptoms (e.g., I feel upset when I cannot access the Internet), mood alteration (e.g., Going online makes me feel better when I am depressed), social comfort (e.g., I feel safer while communicating with others through the Internet), and negative outcomes (e.g., I have some difficulty with school performance because I spend too much time on the Internet). The item was evaluated in the extent of agreement using a five-point scale from 1 (never true) to 5 (always true). This scale was excellent in reliability and validity, Cronbach’s alpha coefficients were more than 0.95 in previous studies (Lei and Wu, 2007; Lei and Yang, 2007; Li and Zheng, 2010; Sun et al., 2015). In the present study, the Cronbach’s alpha coefficients were set to 0.947 for the total scale.

##### Priming words and target images

Priming words enclosed self-related and other-related categories. Self-related words included ‘self, mine, I am, I, etc., and others-related words included ‘other, others, his/her, he/she is, and he/she. The validity of the priming word was tested in previous research (Jiang et al., 2008; He and Zhu, 2016).

Target images were self-made, the preparation was showed as follows. First, undergraduate and graduate students who frequently used Internet were interviewed by face to face, and there were two common online positions: (1) playing cellphone with sitting and standing; (2) playing computer with sitting. Then, an art professional student was invited to draw these pictures that described these events. Finally, there were six pictures regarding playing cellphone with standing and eight pictures regarding playing cellphone and computer with standing and sitting. All pictures were made into images by Photoshop CS5, the images with sitting had same size (701 × 750 pixels), the images with standing had same size (302 × 748 pixels) and all the pictures are the same in resolution (300 dpi). The versions of boy and girl are different in the figures not in the content. Each picture was modified into three different colors in frames, red, blue, and yellow.

#### Procedure

There was 2 (priming type: self, other) × 2 (subject type: pathological-tendency Internet user, ordinary Internet user) mixed design. The within variable was priming type, between-variable was subject type, the dependent variables were the RT and accuracy.

**FIGURE 1:**
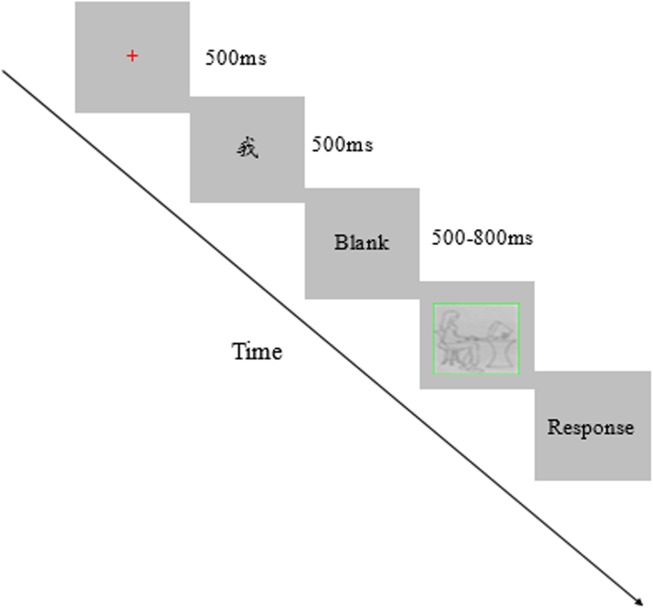
Sequence of events in an experimental trial.

The participant was seated in a quiet room at approximately 60 cm from the computer screen with horizontal and vertical visual angles below 5°. E-prime 2.0 was used to present the stimuli and collect the behavioral data. Before the normal experiment, a practice block with ten trials was arranged for the subject to familiarize the procedure. There were two blocks in the normal experiment, each block comprised 72 trials. Trials were presented randomly in each block, and several minutes were set for the rest between blocks. The self-priming stimuli and other-priming stimuli are presented in one block 36 times, respectively. For each trial, a red fixation was presented 500 ms in the center of the black screen, and the priming stimulus was presented 500 ms, then a blank screen was presented 500–800 ms, followed one of the online images for infinite until the subject pressed the key. A blank screen remained 1000 ms before the new trial started. Participants were asked to judge which color the image frame was, pressing the “d” key if the frame was red, pressing the “g” key if the frame was green, pressing the “J” key if the frame was yellow (see Figure 1).

#### Statistical Analyses

Separate two-way repeated measures of ANOVA were conducted on the RT and accuracy. ANOVA factors included priming type (self, other), subject group (pathological-tendency Internet user, ordinary Internet user). The degrees of freedom of the *F*-ratio were corrected according to the Greenhouse-Geisser method.

## RESULTS

**FIGURE 2:**
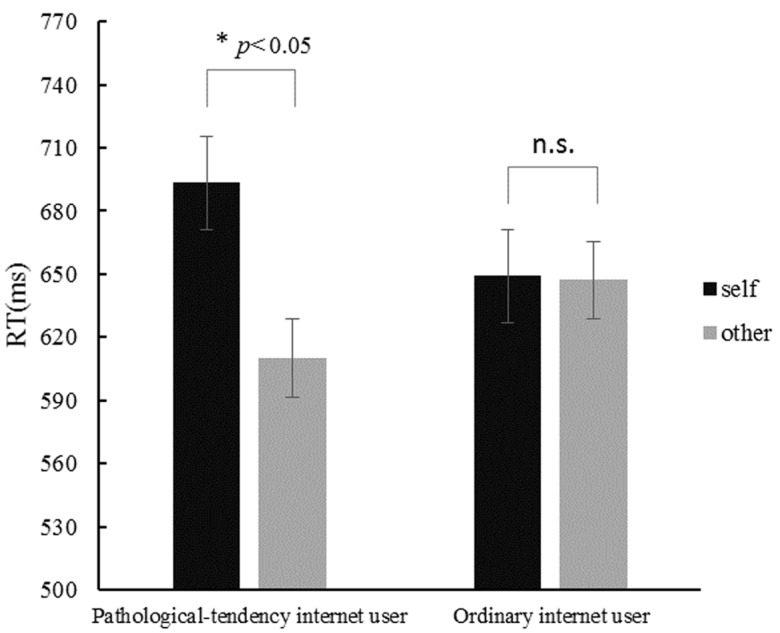
Mean RT in the self priming and other priming conditions in pathological-tendency Internet group and ordinary Internet group. Error bars are SE of the means. Asterisks indicate the significant difference at ^∗^*p* < 0.05 level, ns, no significant difference.

Repeated measures of ANOVA on RT showed a significant main effect of priming type, *F*(1, 40) = 5.83, *p* < 0.05,$\eta_{p}^{2}$ = 0.13. Further analysis showed that longer RT was found under the self-priming condition (671.21 ± 18.62 ms) compared to that under the other-priming condition (628.56 ± 19.60 ms). Furthermore, results also showed significant interaction effect of priming type and subject group, *F*(1, 40) = 5.306, *p* < 0.05, $\eta_{p}^{2}$ = 0.117. Simple effect analysis on the interaction effect of priming type and subject group showed that longer RT was reported under the self-priming condition as compared to the other-priming condition for the pathological-tendency Internet users, *F*(1, 40) = 10.16, *p* < 0.01, but not for the ordinary Internet users. Mean RT under the self-priming and other-priming conditions for pathological-tendency and ordinary Internet users were shown in Figure 2. Similar repeated ANOVAs on accuracy were conducted, and the results showed no main or interaction effects. Mean correct rate in the self-priming and other-priming conditions for pathological-tendency Internet user is 95.3 and 96.2%, respectively. Mean correct rate under the self-priming and other-priming conditions for ordinary Internet users is 96.4 and 96.9%, respectively.

### DISCUSSION

This experiment examined the effects of the Internet use on the self-related process at a behavioral level, and the results showed that the RT under the self-related priming condition was longer than that of the other related priming condition for pathological Internet users, but not for ordinary Internet users. This finding suggested that Internet use significantly influenced the self-related process probably because of the following points: First, Dong et al. (2010) found that pathological Internet users need to invest numerous cognition resources to process target stimuli in inhibition task, reflecting that pathological-tendency Internet users have low inhibition ability. In our study, our experimental design is similar to the inhibition task. The Internet information denotes distract stimuli, and the color of frame denotes target stimuli. Low inhibition ability for pathological Internet users allows them to have longer RT in Experiment 1. Second, pathological Internet users play attention bias toward the Internet information because Internet-related information elicited a self-schema of the pathological Internet users (Zhou et al., 2012; Näsi and Koivusilta, 2013).

Although pathological-tendency Internet users showed different behavioral responses to process the information of playing with a cellphone or a computer from ordinary Internet users, the RT index could not fully capture the self-related process in an Internet context. Therefore, we performed Experiment 2 to reveal the neural correlation of the self-related process in the Internet context.

## Experiment 2

### Methods

#### Participants

A larger-scale online survey was conducted to recruit the participants. There are 1000 college students completed the APIUS. Based on the same criterion to experiment 1, 25 pathological-tendency Internet users and 24 ordinary Internet users, aged 18–26 years were recruited in this experiment. In order to test the reliability of the criterion, 49 participants were asked to complete the APIUS again a week later. Four subjects were removed from pathological-tendency Internet users for their two mean score were less than 114 points, and three subjects were removed from ordinary Internet users because of problems during recording ERP data. Finally a total of 42 Chinese undergraduate students (21 pathological-tendency Internet users: *M* = 131.45, *SD* = 13.88, 21 ordinary Internet users: *M* = 81.76, *SD* = 15.78) were used for data analysis. They were 18–26 years (*M* = 20.86, *SD* = 2.18) and healthy, right-handed, with normal or corrected to normal vision, with no history of brain injuries or affective disorders by self-reported. Each participant was paid RMB 30 Yuan after they completed the experiment. There is no subject participated in both experiment 1 and experiment 2.

#### Ethics

The present study was approved by the Central China Normal University Human Ethics Committee for Brain Mapping Research, and written informed consent was obtained from each participant before experiment.

#### Measurements

The scale and materials were the same as in the Experiment 1. In the Experiment 2, the Cronbach’s alpha coefficient of APIUS was 0.955 in the total scale for the first time, and 0.958 in the total scale for the second time.

#### Procedure

There was 2 (priming type: self, other) × 2 (subject type: pathological-tendency Internet users, ordinary Internet user) mixed design. The within variable was priming type, between-variable was subject type, and the dependent variables were RT, accuracy, the amplitude of N2 and LPC. The procedure was the same as the experiment 1.

#### ERP Recordings and Analysis

Electroencephalograms (EEGs) were recorded by 64 scalp silver/silver-chloride electrodes according to the international 10–20 system. All electrodes were referenced to an electrode at the left mastoid and re-referenced off-line to the bilateral mastoid (Luck, 2014). The horizontal electrocardiogram (EOG) was recorded in a bipolar manner from two electrodes placed 1.5 cm lateral to the left and right outer Chianti, and the vertical EOG was recorded from electrodes below and above the left eye. The impedance for each electrode was kept below 5 kΩ. EEG was amplified (half-amplitude band pass 0.05–100 Hz) and digitized at a sampling rate of 500 Hz for each channel.

Using the open source toolkit EEGLAB in the MATLAB environment to process ERP data (Delorme and Makeig, 2004), horizontal and vertical electrooculogram electrodes and data were excluded. The bilateral mastoid was used as a reference for analysis. The filter band pass was 0.01–30 Hz, and ICA was used to remove artifacts such as myoelectricity, blinking, and eye movements, etc. Based on prior research and grand average waves, the analysis intervals began at an average of 200 ms prior to the stimuli and ended at 1000 ms after the onset of the stimulus. The baseline was the 200 ms before the onset of the priming stimuli. The time window for the N2 component and LPC component are based on the previous literatures (N2: Chen et al., 2011, 2013; Fan et al., 2016. LPC: Zhong et al., 2014; Yuan et al., 2018) and the grand averaged ERPs in the present study. The average amplitudes of the frontal N2 components (230–280 ms) and the mean amplitude of the frontal late LPC component (550–1000 ms) (Zhong et al., 2014; Kotlewska and Nowicka, 2016) were analyzed. These electrodes analyzed were Fz, FCz, and Cz. Statistical analysis was performed by the SPSS 20.0 statistical analysis software package. All variance analysis *p*-values were corrected using the Greenhouse-Geisser method.

### Results

#### Behavioral Results

Repeated measures of ANOVA on the RT showed main effects of subject group [*F*(1, 40) = 4.105, *p* < 0.05,$\eta_{p}^{2}$ = 0.093] and priming type [*F*(1, 40) = 5.325, *p* < 0.05,$\eta_{p}^{2}$ = 0.117]. Further analysis revealed that pathological-tendency Internet users (567.52 ± 25.42) respond faster than ordinary Internet users (640.36 ± 25.42), RT in self-related condition (608.32 ± 18.60) was longer than that of other-related condition (599.55 ± 17.53).

Similar repeated measures of ANOVA were conducted on the accuracy, results showed that there was a significant main effect of subject group, *F*(1, 40) = 5.41, *p* < 0.05, $\eta_{p}^{2}$ = 0.119. Further analysis showed that ordinary Internet users (0.975 ± 0.005) performed more accurately than pathological-tendency Internet users (0.958 ± 0.005). Furthermore, results also showed a significant interaction effect of priming type and subject group, *F*(1, 40) = 5.62, *p* < 0.05, $\eta_{p}^{2}$ = 0.123. Simple effect analysis on the interaction effect of priming type and subject group showed that the accuracy in self priming condition was higher than that of other priming condition for pathological-tendency Internet users [*F*(1, 40) = 10.16, *p* < 0.01], but not for ordinary Internet users.

#### ERP Results

**FIGURE 3:**
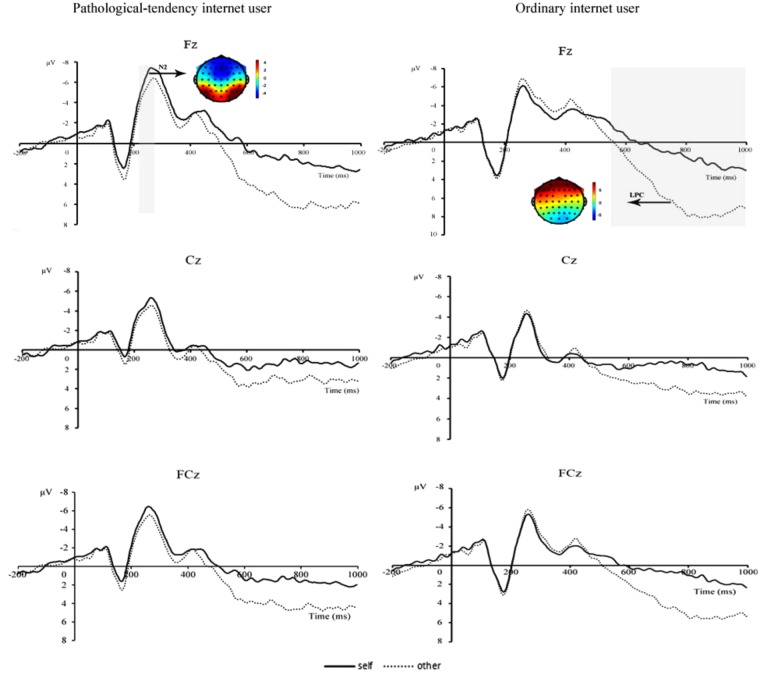
Averaged ERPs at Fz, Cz, and FCz, for two priming condition in pathological-tendency Internet user and ordinary Internet user.

As shown in Figure 3 N2 and LPC components were elicited under both self-related and other-related priming conditions for pathological-tendency Internet group and ordinary Internet group. Separate three-way repeated measures analyses of variance (ANOVAs) were conducted on each component. The factors were priming type (self, other), subject type (pathological-tendency Internet users, ordinary Internet users), and electrode (Fz, Cz, and FCz). The degrees of freedom of the *F*-ratios were corrected by Greenhouse-Geisser method.

##### N2 (230–280 ms)

**FIGURE 4:**
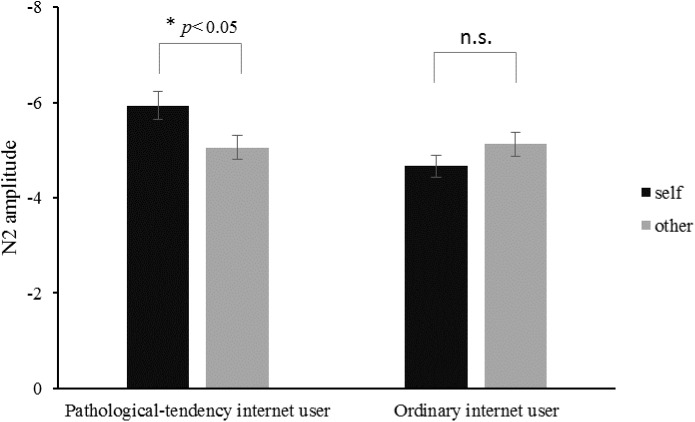
Mean amplitudes of the N2 components in the self priming and other priming conditions in pathological-tendency Internet user and ordinary Internet user. Asterisks indicate the significant difference at ^∗^*p* < 0.05 level, ns, no significant difference.

Three-way repeated measures of ANOVA were conducted on the N2 amplitudes, results showed significant interaction effect of priming type and subject group, *F*(1,40) = 4.758, *p* < 0.05, $\eta_{p}^{2}$ = 0.106. Simple effect analysis on the interaction effect of priming type and subject group showed that the self priming had larger N2 amplitudes as compared with the other priming in pathological-tendency Internet user *F*(1, 40) = 4.12, *p* < 0.05. Mean amplitudes of the N2 components in the self priming and other priming conditions in pathological-tendency Internet group and ordinary Internet group were shown in Figure 4.

##### LPC (550–1000 ms)

Three-way repeated measures of ANOVA were conducted on the mean amplitude of the LPC, results showed there was a significant main effect of priming type, *F*(1,40) = 15.27, *p* < 0.001,$\eta_{p}^{2}$ = 0.276. Further analysis found smaller LPC amplitudes were showed in self-related priming condition (1.27 ± 1.11) as compared to other-related priming condition (4.27 ± 1.27).

#### Discussion

The results showed that the accuracy of the pathological-tendency Internet users was lower than that of the ordinary Internet users. This finding suggested that excessive Internet use impairs an individual’s ability to process information (Xiong and Yao, 2010; Näsi and Koivusilta, 2013), especially self-control (Mei et al., 2016). Pathological Internet users are easily distracted by irrelevant information, but one’s cognitive resources are limited (Baumeister et al., 2007). Therefore, their accuracy is lower than that of ordinary Internet users when more cognitive resources are needed to inhibit the interference of unrelated events in the judgment task (Campanella et al., 2002).

Moreover, more negative front–central N2 amplitudes existed under the self-related priming condition than under the other related priming conditions. The N2 component is related to inhibition control or conflict monitoring (Buzzell et al., 2014). Thus, participants need more cognitive resources to inhibit the interference of unrelated events in processing self-related priming stimuli in early phases than that of in processing other related priming stimuli because the former is more important to one’s self than the latter (Campanella et al., 2002). This study also showed that the LPC amplitudes elicited by the self-related priming stimuli were smaller than those of the other related priming stimuli. The LPC component indicated that the continuing cognitive resource poured the judgment task, and it is sensitive in evaluating the classification (Liu et al., 2013). Therefore, individuals distributed less cognitive resources on the self-related information processing relative to the other related information and automatically activated the self-related scheme to good judgment.

## General Discussion

To our knowledge, the current study is the first to investigate the self-related process in the Internet context. Two experiments tested the effect of Internet use on the self-related process in terms of behavior and neural correlations.

Consistent with prior findings, behavioral results showed that participants required a long RT to judge the frame’s color under the self-priming condition. For example, individuals focus on and deliberately process self-related stimuli (Fan et al., 2016). Self-related information was more important than other related information and should be prioritized in cognitive processing. However, the behavioral results from Experiment 2 were inconsistent, that is, they revealed that the RT of the pathological Internet users was shorter than that of the ordinary Internet users, and the accuracy of the former was lower than that of that latter. PIU belonged to compulsive-impulsive spectrum disorder (Zhou et al., 2012). Thus, a cognitive bias similar to pathological gambling, drug addiction, or alcohol abuse exists (Noël et al., 2005; Linnet et al., 2012), that is, pathological Internet users should have longer RT and lower accuracy in cognitive tasks than those of the ordinary Internet users. However, we cannot find the cognitive bias in RT, and this study cannot explain it reasonably. As such, this parameter should be investigated further.

N2 can reflect the individuals’ inhibited unrelated information; the N2 amplitude was larger under the self-related condition than that of under the other related conditions for pathological-tendency Internet users, but not for ordinary Internet users. This finding is consistent with the idea that pathological Internet use impairs the cognition process (Pawlikowski and Brand, 2011; Näsi and Koivusilta, 2013). Pathological Internet users have a deficient inhibitory control in the N2 component (Zhou et al., 2010). Excessive computer game players also have a lower inhibition behavior than typical computer user (Littel et al., 2012). Hence, pathological Internet users possibly are deficient in inhibition ability, so they need more cognition resources to exclude the distraction of Internet-related information (online pictures) to judge the color of the target frame. This study further supported that excessive Internet use reduced the cognitive ability in the self-related process.

Furthermore, LPC under the self-related priming condition was smaller than that of the other related condition. LPC can reflect individuals’ self-related process in implicit priming task (Kok, 1997), so this finding indicated that cognitive resources are not considerably needed in self-related information processes (Fields and Kuperberg, 2016). This result is inconsistent with previous findings, that is, processing self-related cues elicited larger LPC than other related cues in self-reference task. The first reason is that self-related information process was inhibited in the Internet context (Yuan et al., 2018), especially pathological Internet users. Moreover, small LPC demonstrated that cognitive resource was not highly needed in an automatic process. Another explanation was that all of the participants were familiar with the Internet context. Both pathological Internet users and ordinary Internet users can automatically process the self-related online stimuli. Hence, Internet-related experiences probably changed the self-schema developed in the real world. However, further studies should be performed to confirm this view.

The present study adopted the implicit priming task to explore the self-related process in the Internet context and found that Internet use influenced the self-related process at both behavioral and neural levels. This study extended the self-related theory and helped in intervening Internet addicts. However, several limitations exist. First, this study only examined the individual aspect of self. Future research should examine whether it can be generalized to other aspects of self, such as collective and ethnic self-related processes in the Internet context. Second, Internet use comprised different contents, such as online game and information searching, and each category might influence the self-related process differently. Future research should examine the differences in self-related processing. Finally, findings should be test again by longitudinal design with a larger sample.

## Conclusion

Internet use influenced the self-related process, especially in inhibition control and automatically retrieving the Internet-related information.

## Author Contributions

FK designed the experiments and revised the paper. GZ collected and analyzed the data and wrote the paper. ZL, YW, BZ, XZ, and FT recruited participants and collected data. ZZ and YZ revised the paper. All authors agreed on the final version of the manuscript.

## Conflict of Interest Statement

The authors declare that the research was conducted in the absence of any commercial or financial relationships that could be construed as a potential conflict of interest.
